# Comprehensive Assessment of the Relationship Between MicroRNA-124 and the Prognostic Significance of Cancer

**DOI:** 10.3389/fonc.2018.00252

**Published:** 2018-07-16

**Authors:** Yadong Sun, Fujiao Duan, Weigang Liu, Zhen Peng, Liping Dai, Yajing Feng, Zhenxing Yang, Jia Shang, Kaijuan Wang

**Affiliations:** ^1^Department of Breast and Medical Research Office, Affiliated Cancer Hospital of Zhengzhou University, Zhengzhou, China; ^2^College of Public Health, Zhengzhou University, Zhengzhou, China; ^3^Medical Record Statistics Office, Affiliated Hospital of Hebei University of Engineering, Handan, China; ^4^Department of Infectious Disease, People’s Hospital of Zhengzhou University, Henan Provincial People’s Hospital, Zhengzhou, China; ^5^Department of Nosocomial Infection Management, The First Affiliated Hospital of Zhengzhou University, Zhengzhou, China

**Keywords:** miR-124, prognosis, cancer, risk factor, comprehensive assessment

## Abstract

**Background:**

Numerous studies have demonstrated the presence of microRNA-124 abnormalities involving gene expression, methylation, and single nucleotide polymorphism (SNP) in multiple and diverse cancers, but the prognostic value of these abnormalities in cancer remains inconclusive.

**Objective:**

The aim of this study is to determine the prognostic value of miR-124 in cancer.

**Methods:**

We scrutinized the electronic databases and estimate the association between miR-124 expression, methylation and single nucleotide polymorphisms (SNPs), and prognosis in cancers. The pooled hazard ratios with 95% confidence intervals (CIs) for overall survival (OS), and disease-free survival/recurrence-free survival (RFS)/progression-free survival (PFS) were calculated to estimate the effects of miR-124 expression, methylation, and SNPs on cancer prognosis. The Quality in Prognosis Studies and Newcastle-Ottawa Scale were utilized to assess the quality of included studies.

**Results:**

A total of 20 studies involving 3,574 participants were analyzed in evidence synthesis. Our findings showed that the low expression of miR-124 was significantly associated with poor OS (HR = 2.37, 95% CI: 1.91–2.94, *P* = 0.00; HR = 3.10, 95% CI: 2.04–4.70, *P* = 0.00) and PFS/RFS (HR = 2.21, 95% CI: 1.50–3.26, *P* = 0.00; HR = 2.12, 95% CI: 1.20–3.74, *P* = 0.00). The hyper-methylation of miR-124 was associated with poor OS (HR = 2.09, 95% CI: 1.48–2.95, *P* = 0.00) and PFS (HR = 3.70, 95% CI: 1.72–7.97, *P* = 0.00) (Table 3). The patients carrying with Allele C of miR-124 rs5315649 had a worse OS (HR = 1.50, 95% CI: 1.09–2.07, *P* = 0.00) and PFS (HR = 1.67, 95% CI: 1.20–2.33, *P* = 0.00) than the carriers with Allele G.

**Conclusion:**

The low expression and hyper-methylation of miR-124 was strongly associated with poor prognosis, and genetic variations of miR-124 rs531564 affected prognosis in cancer patients.

## Introduction

MicroRNAs (miRNAs) are small, non-protein-coding RNA molecules involved in RNA silencing and posttranscriptional regulation of gene expression (1, 2). Numerous studies have proved that abnormalities of miRNAs are involved in various cancers, which play important roles in many aspects of carcinogenesis and act as oncogenes or tumor suppressors, including cell differentiation, proliferation, angiogenesis, and metastasis (3–6).

MicroRNAs regulate genes expression by binding to the 3′-untranslated region of target mRNAs (7, 8). Given the high stability of miRNAs in formalin-fixed, paraffin-embedded tissue and circulation, they are increasingly considered as biomarkers for predicting cancer prognosis and treatment response (9–11). Previous studies have demonstrated that miRNAs are aberrantly expressed in various types of cancer and involved in different biological processes, such as differentiation, cell growth, migration, and apoptosis (12).

Human microRNA-124 (miR-124) is encoded by three loci: miR-124-1 (8p23.1), miR-124-2 (8q12.3), and miR-124-3 (20q13.33) (13). MiR-124 is significantly downregulated in various tissues and cell lines of cancer. Overexpression of miR-124 suppresses migration, cell proliferation, and invasion and induces apoptosis by regulating Rac1, indicating that miR-124 plays a tumor suppressive role in various cancer (14–16). It had been demonstrated in diverse cancer types, such as non-small cell lung cancer (NSCLC) (16), hepatocellular carcinoma (17), glioblastoma multiforme (18), gastric cancer (19), ovarian cancer (20), breast cancer (21–24), and colorectal cancer (25). However, little is known about the association between the cancer prognosis and expression levels of miR-124 in tissues or serum.

Aberrant DNA methylation of promoter CpG islands permanently inactivates tumor suppressor genes and is profoundly involved in carcinogenesis, similar to chromosomal abnormalities and mutations (26). Downregulation of miR-124 by promoter methylation has been observed in gastric cancer (27), colorectal cancer (28), prostate cancer (29), cervical and pancreatic cancers (13). Methylation-mediated downregulation of MiR-124 can be observed in 85% of lung cancer patients (30). As a novel risk marker for cancer, the methylation levels of miR-124 and the epidemiological risk of cancer patients need to be specified.

Common genetic polymorphisms in miRNAs and miRNA-processing pathway genes are well established in tumor development and progression (31). Single nucleotide polymorphisms (SNPs) in miRNA-processing pathway genes or miRNAs may alter the transcription and expression of miRNAs and are, therefore, associated with the risks and outcomes of various cancers (32). Since SNPs associated with the risk of cancer may affect prognosis, analysis of relevant SNPs in miRNAs may help to find novel cancer therapeutic targets and prognostic biomarkers (33).

To date, there is no available information on system-based evidence-based medicine for the prognostic value of miR-124. Furthermore, the role of miR-124 in cellular proliferation and invasion of cancer is not fully understood. Numerously, previous studies have few new or insightful arguments in their reports that contributed significantly to the field of cancer biology. Therefore, the prognostic data of miR-124 need to be assimilated from different studies to draw the conclusion. In this study, we used quantitative synthesis to precisely quantify the expression, methylation levels, and SNP (rs5315649) of miR-124 to assess the prognostic significance in cancer patients.

## Materials and Methods

### Search Strategy

This study was executed in accordance with criteria of Meta-analysis of Observational Studies in Epidemiology group (MOOSE) (34) and the Preferred Reporting Items for Systematic Reviews and Meta-analysis (PRISMA) (35). The protocol of this meta-analysis has not been published or registered to any databases.

We scrutinized the following electronic databases until December 2017: PubMed, Web of Science, Google Scholar, Embase, Wanfang medicine online, and Chinese National Knowledge Infrastructure (CNKI). The search strategy was set up using the key words: “carcinoma” or “cancer” or “tumor” and “microRNA-124” or “miR-124” and “Methylation” and “polymorphisms” and “prognosis” or “survival” or “outcome” in humans. We also manually searched reference lists of relevant articles to further identify potential studies that not retrieved by databases exploration.

Subsequently, citations selected from initial search were screened for eligibility by two authors independently (Fujiao Duan and Zhenxing Yang). Articles that met all selection criteria were retrieved.

### Inclusion and Exclusion Criteria

The including criteria were: (i) cohort studies that investigated the relationship between miR-124 and prognostic indicators including overall survival (OS) and/or progression-free survival (PFS)/recurrence-free survival (RFS)/disease-free survival (DFS) of cancer patients; (ii) the expression levels of miR-124 was measured in cancer tissue or serum; (iii) hazard ratios (HRs) and corresponding 95% CIs for survival analysis were reported in studies or could be computed from given data; (iv) available in Chinese or English language.

The exclusion criteria were: (i) studies that were not conducted in cancer patients; (ii) neither Chinese nor English language; (iii) review articles, case reports, or letters; (iv) with insufficient data to calculate the HRs and their 95% CIs, or the Kaplan–Meier curve unable to calculate HRs and 95% CI parameters.

Duplicate publications were eliminated through the Mendeley software (36). If a study had overlapping data with other published literatures, we selected the study with a larger sample size or the latest published article. All targeted articles were then evaluated and screened for eligibility by two reviewers (Zhen Peng and Weigang Liu) independently, and conflicts were finalized after consultation with third author.

### Methodological Quality Assessment

When the prognostic result was reported only as the Kaplan–Meier curves in some studies, the Engauge Digitizer 4.1 was then used to obtain the survival data, and Tierney’s method to calculate the HRs and their 95% CIs (37). The quality of the enrolled studies was assessed by the Newcastle-Ottawa Scale (NOS). The NOS consists of three quality parameters with a total of 9 points. Studies with a NOS score greater than 6 were considered as high-quality.

The specific Quality in Prognosis Studies (QUIPS) for specific biases of prognosis was appraised based on the approach of Hayden et al. (38). Estimation of the potential bias of the items included study participation, study attrition, prognostic factor measurement, outcome measurement, study confounding, statistical analysis, and reporting.

Two reviewers (Fujiao Duan and Zhenxing Yang) performed the quality assessments separately and, in case of any inconsistency, the final decision was reached with consensus.

### Statistical Analysis

The HR with 95% CI was used to evaluate the impact of miR-124 expression on of cancer patients. Inter-study heterogeneity was quantified using *Q*-tests and the *I*-squared (*I*^2^) test (39). In the absence of significant heterogeneity (*P*_heterogeneity_ > 0.10 or *I*^2^ < 50%), a fixed-effects model (Mantel–Haenszel method) (40) was appropriately used to calculate the pooled effect, otherwise, the random-effects model (DerSimonian and Laird method) (41) was employed, and meta-regression was further utilized to explore sources of heterogeneity (42).

Begg’s funnel plot (rank correlation test) (43) and Egger’s test (44) determined the potential publication bias among included studies. One-way sensitivity analyses were performed, and then by omitting each study in turn to examine the stability of the pooled results.

All statistical analyses were performed with RevMan (Version 5.3.5 for Windows, Cochrane Collaboration, Oxford, UK) and Stata 13.1 MP (Stata Corporation, College Station, TX, USA). A two-tailed value of *P* < 0.05 was considered statistically significant.

## Results

### Study Identification

**Figure 1:**
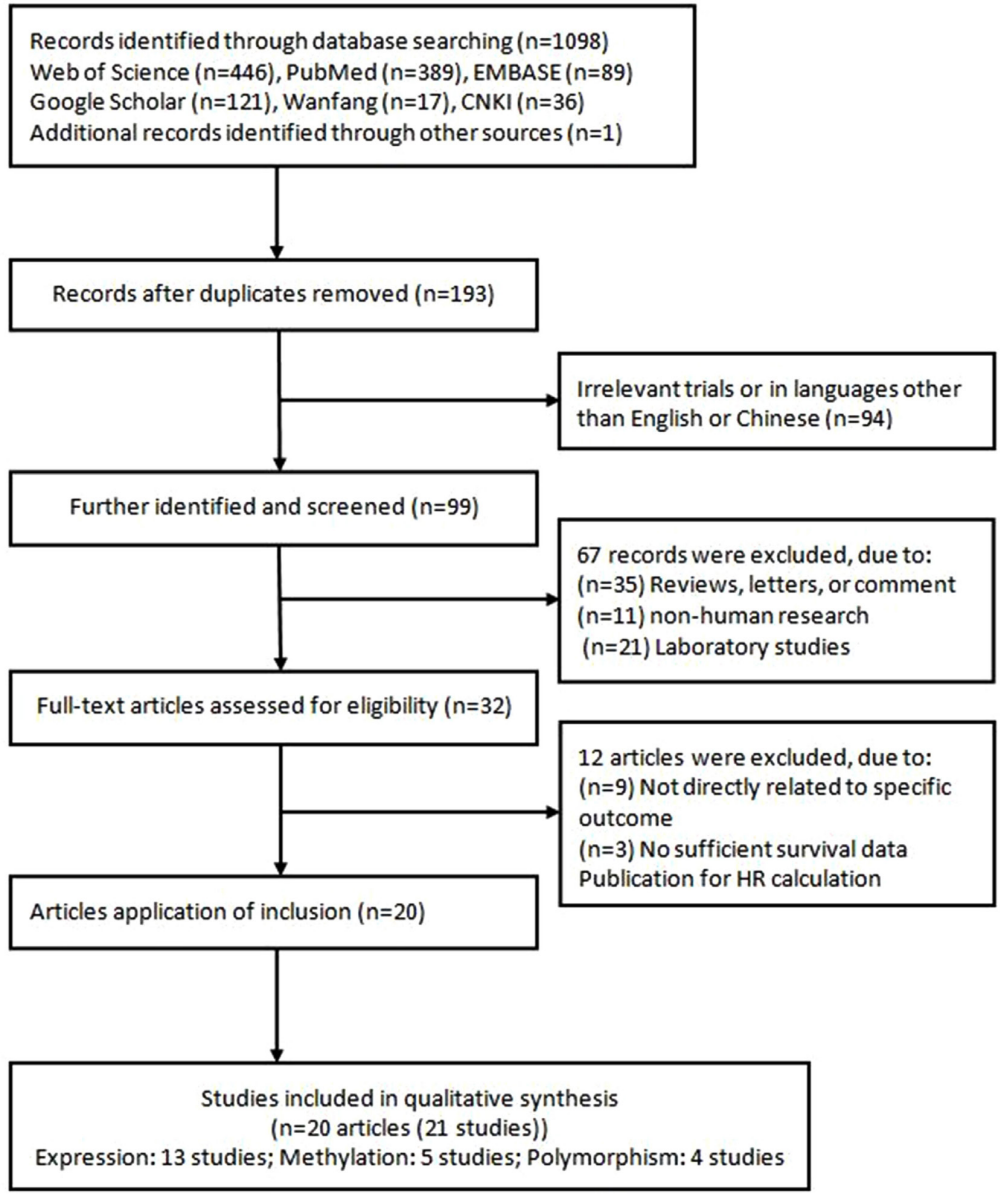
Flow chart of literature search and study selection.

The systematic search returned 1,098 publications based on the search strategy (Figure 1). According to the exclusion criteria, the abstracts of 193 studies were reviewed. Of them, 94 were excluded because of irrelevant trials or in languages other than English or Chinese; 68 were excluded because they were reviews, letters, comments, non-human research, or laboratory studies. Eventually, 32 articles were eligible for further analysis. However, 12 articles were excluded as they were not directly related to specific outcome or they had insufficient survival data published for a HR calculation. Therefore, 20 articles (30, 45–63) (21 studies) were finally included in the meta-analysis. One of the articles (61) performed two cohorts in different populations, and we considered it as two studies.

### Baseline Characteristics of Included Studies

The major characteristics of eligible studies are summarized in Table 1. The studies were published from 2013 to 2017 and included a total of 3,574 patients from China, Iran, Japan Korea, Germany, and Canada. The patients were classified as Asian or Caucasian according to their ethnic background. The types of cancer included colorectal cancer, gastric cancer, osteosarcoma, pancreatic ductal adenocarcinoma (PDAC), breast cancer, NSCLC, glioma, renal cell cancer, acute myelocytic leukemia, pancreatic cancer, and esophageal adenocarcinoma. The method of miR-124 detection was quantitative real-time polymerase chain reaction (qRT-PCR), quantitative methylation-specific real-time PCR analysis (qMSP), and polymerase chain reaction ligase detection reaction (PCR-LDR) in 21 studies. MiR-124 expression, methylation levels, and rs5315649 for OS and/or DFS/RFS/PFS were measured in tissue or serum. The cutoff values of miR-124 were different in the studies, with most taken as the median.

**Table 1:** Clinicopathological characteristics of eligible studies.

Study	Year	Country	Expression		Methylation		rs5315649		Histology	TNM stage	Sample	Assay	Follow-up (months)	Cut-off	Outcome
			OS	Other	OS	Other	OS	Other							
Cong et al. (45)	2018	China	114	DFS, 114					Osteosarcoma	I–III	Serum	qRT-PCR	60	Median	HR/SC
Margolinmiller et al. (46)	2017	Israel	67	PFS, 67					Ependymoma	NA	Frozen tissue	qRT-PCR	232	Median	HR/SC
Liu et al. (47)	2016	China	126	DFS, 126					Gastric cancer	I–IV	Frozen tissue	RTFQ-PCR	58	Normal	SC
Sun et al. (48)	2016	China	53						PDAC	I–III	Serum	qRT-PCR	147	Median	HR/SC
Ali et al. (49)	2015	Iran	100						Breast cancer	I–III	Frozen tissue	qRT-PCR	49	Median	HR
Dong et al. (50)	2015	China	133						Breast cancer	I–III	Frozen tissue	qRT-PCR	60	Median	HR/SC
Li et al. (51)	2015	China	164	DFS, 164					NSCLC	I–III	Frozen tissue	qRT-PCR	50	Normal	HR/SC
Lv et al. (52)	2015	China	71	PFS, 71					Colorectal cancer	II–IV	Frozen tissue/serum	qRT-PCR	92	Normal	HR/SC
Chen et al. (53)	2015	China	137	PFS, 137					Glioma	I–IV	Frozen tissue	qRT-PCR	60	Normal	HR/SC
Zhang et al. (54)	2015	China	92	DFS, 92					NSCLC	I–IV	Frozen tissue	qRT-PCR	60	Median	HR/SC
Jinushi et al. (55)	2014	Japan	49	PFS, 49					Colorectal cancer	I–IV	Frozen tissue/serum	qRT-PCR	95	Median	HR/SC
Wang et al. (56)	2013	China	96	DFS, 96					Colorectal cancer	I–IV	Frozen tissue	qRT-PCR	52	Normal	HR
Wang et al. (57)	2017	China			56	PFS, 34			AML	NA	Serum	qMSP	48	Median	HR/SC
Kim et al. (30)	2016	Korea			157				NSCLC	I–III	Frozen tissue	qMSP	120	Normal	HR/SC
Peters et al. (58)	2014	Germany			18	PFS, 18			Renal cell cancer	NA	Frozen tissue	qMSP	60	Median	HR/SC
Wang et al. (59)	2014	China	65		65				Pancreatic cancer	I–IV	Frozen tissue	qMSP	60	Median	HR/SC
Gebauer et al. (60)	2013	Germany				PFS, 111			Renal cell cancer	I–III	Frozen tissue	qMSP	70	Median	HR/SC
Faluyi et al. (61)	2017	Canada					231	PFS, 231	EA	I–III	Serum	SNaPShot	72	Median	HR/SC
	2017	Canada					137	PFS, 137	EA	I–III	Serum	SNaPShot	72	Median	HR/SC
Shi et al. (62)	2016	China					174		Osteosarcoma	I–III	Serum	PCR-LDR	60	Median	HR/SC
Ying et al. (63)	2016	China						RFS, 1358	Colorectal cancer	I–III	Serum	MassARRAY	36	Median	HR/SC

*NSCLC, non-small cell lung cancer; PDAC, pancreatic ductal adenocarcinoma; AML, acute myelocytic leukemia; EA, esophageal adenocarcinoma; qRT-PCR, quantitative real-time PCR; OS, overall survival; PFS, progressive free survival; DFS, disease-free survival; RFS, recurrence-free survival; SC, survival curve. qMSP, quantitative methylation-specific real-time PCR analysis; PCR-LDR, polymerase chain reaction ligase detection reaction*.

### Qualitative Assessment

**Figure 2:**
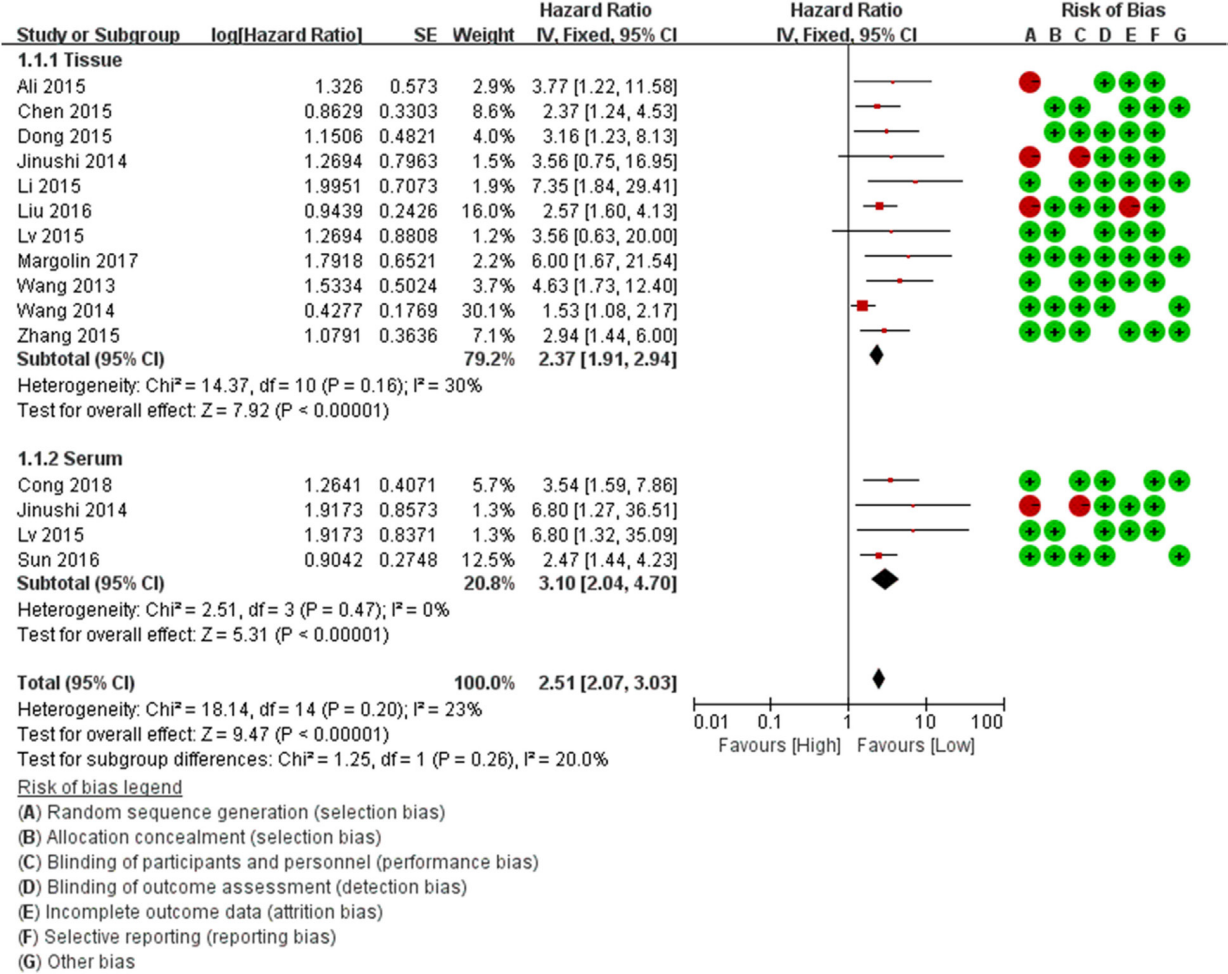
Forest plots of studies evaluating the hazard ratios of miR-124 expression (tissue and serum) with respect to overall survival.

**Figure 3:**
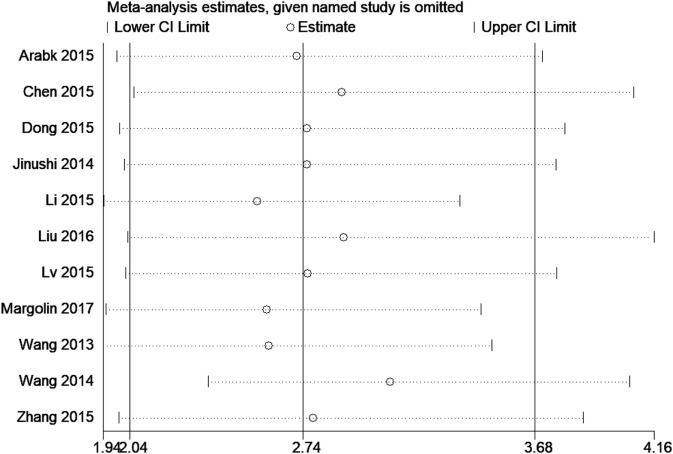
Sensitivity analysis for overall survival (tissue) of miR-124.

The result of quality assessment of the included studies based on QUIPS was summarized in Table 2. The bias domains of estimated items include participation, attrition, measurement of prognostic factor, confounding measurement and account, outcome measurement, and analysis and reporting. The risks of bias legend were presented in Figures 2 and 3. Based on the NOS (Table A1 in Appendix), 70 percent (14/20) of the enrolled studies were high-quality (quality score ≥ 6).

**Table 2:** Quality assessment of included studies based on the quality in prognosis studies.

Study	Quality evaluation of prognosis study						Total score^a^	Level of evidence^b^
	Study participation	Study attrition	Prognostic factor measurement	Outcome measurement	Study confounding	Statistical analysis and reporting		
Cong et al. (45)	Yes	Partly	Partly	Yes	Partly	Yes	7	2b
Margolinmiller et al. (46)	Yes	Yes	Yes	Yes	Partly	Yes	9	1b
Liu et al. (47)	Partly	Partly	Partly	Partly	Partly	Partly	6	2b
Sun et al. (48)	Yes	Partly	Partly	Yes	Partly	Partly	4	2b
Ali et al. (49)	Yes	Partly	Partly	Partly	Partly	Partly	5	2b
Dong et al. (50)	Yes	Partly	Yes	Partly	Partly	Partly	6	2b
Li et al. (51)	Yes	Partly	Yes	Yes	Partly	Yes	7	2b
Lv et al. (52)	Yes	Partly	Partly	Yes	Partly	Yes	7	2b
Chen et al. (53)	Partly	Partly	Yes	Yes	Partly	Yes	6	2b
Zhang et al. (54)	Yes	Yes	Partly	Partly	Partly	Yes	7	2b
Jinushi et al. (55)	Partly	Partly	Yes	Yes	Partly	Yes	6	2b
Wang et al. (56)	Yes	Partly	Yes	Yes	Partly	Yes	7	2b
Wang et al. (57)	Partly	Partly	Yes	Yes	Partly	Yes	5	2b
Kim et al. (30)	Partly	Partly	Yes	Yes	Partly	Yes	5	2b
Peters et al. (58)	Yes	Yes	Partly	Partly	Partly	Yes	5	2b
Wang et al. (59)	Yes	Partly	Yes	Partly	Partly	Partly	7	2b
Gebauer et al. (60)	Yes	Partly	Yes	Yes	Partly	Partly	6	2b
Faluyi et al. (61)	Yes	Partly	Yes	Yes	Partly	Yes	6	2b
Shi et al. (62)	Yes	Yes	Yes	Yes	Partly	Yes	8	1b
Ying et al. (63)	Yes	Partly	Yes	Partly	Partly	Yes	5	2b

*^a^Quality assessment of included studies based on the Newcastle–Ottawa Scale*.

*^b^The levels of evidence were estimated for all included studies with the Oxford Centre for Evidence-Based Medicine criteria*.

### Meta-Analysis Findings

#### Relationship Between the Expression of mir-124 and Patients’ Survival

For the OS, HRs were provided in 13 studies, and a significant association was observed between low miR-124 level and poor OS in patients (HR = 2.67, 95% CI: 2.10–2.38, *P* = 0.00). We conduct stratified analysis based on different sources, and the results showed that low expression of miR-124 in both serum (HR = 2.37, 95% CI: 1.91–2.94, *P* = 0.00) and cancer tissue (HR = 3.10, 95% CI: 2.04–4.70, *P* = 0.00) was significantly associated with poor OS (HR = 2.37, 95% CI: 1.91–2.94, *P* = 0.00; HR = 3.10, 95% CI: 2.04–4.70, *P* = 0.00). The test results showed that there was no heterogeneity between subgroups (*I*^2^ = 20%, *P* = 0.26) (Table 3; Figure 2).

**Table 3:** Main results of pooled hazard ratios in the meta-analysis.

Comparisons (microRNA-124)	Heterogeneity test			Summary HR (95% CI)	Hypothesis test		Studies
	*Q*	*P*	*I*^2^ (%)		*Z*	*P*	
**Expression**							
Overall survival (OS)							
Total	15.28	0.23	21	2.67 (2.10, 3.38)	3.76	0.00	13
Tissue	14.37	0.16	30	2.37 (1.91, 2.94)	7.92	0.00	11
Serum	2.51	0.47	0	3.10 (2.04, 4.70)	5.31	0.00	4
Subgroup differences	1.25	0.26	20				

**PRS/disease-free survival**							
Total	18.43	0.02	57	3.92 (1.71, 8.96)	4.50	0.00	9
Tissue	15.92	0.03	56	2.21 (1.50, 3.26)	4.00	0.00	8
Serum	3.15	0.21	37	2.12 (1.20, 3.74)	2.59	0.01	3
Subgroup differences	0.01	0.90	0				

**Methylation**							
OS	0.85	0.84	0	2.09 (1.48, 2.95)	4.17	0.00	4
Progression-free survival (PFS)	3.54	0.17	43	3.70 (1.72, 7.97)	2.28	0.00	3

**Polymorphisms**							
OS							
Allele C	0.31	0.58	0	1.50 (1.09, 2.07)	2.50	0.01	2
Dominant model	–	–	–	4.61 (1.85, 11.49)	2.38	0.00	1

**PFS/recurrence-free survival**							
Allele C	0.01	0.98	0	1.67 (1.20, 2.33)	3.06	0.00	2
Dominant model	–	–	–	2.37 (1.36, 4.13)	3.04	0.00	1

*DTC, digestive tract cancer, including colorectal cancer, esophageal squamous cell carcinoma, pancreatic pancer and hepatocellular carcinoma, oral cancer*.

Our analysis revealed a negative correlation between miR-124 level and PFS/RFS (HR = 3.92, 95% CI: 1.71–8.96, *P* = 0.00). Meanwhile, stratified analysis of different sources showed the low expression of miR-124 in serum (HR = 2.21, 95% CI: 1.50–3.26, *P* = 0.00) and cancer tissue (HR = 2.12, 95% CI: 1.20–3.74, *P* = 0.00) was statistically significant with the poor OS respectively. In tests for subgroup differences, the results showed that there was no heterogeneity between subgroups (*I*^2^ = 0%, *P* = 0.90) (Table 3).

#### Relationship Between the Methylation of mir-124 and Patients’ Survival

The results showed that hyper-methylation of miR-124 was associated with poor OS (HR = 2.09, 95% CI: 1.48–2.95, *P* = 0.00) and PFS (HR = 3.70, 95% CI: 1.72–7.97, *P* = 0.00) (Table 3).

#### Relationship Between the SNP of mir-124 and Patients’ Survival

The patients carrying with Allele C of miR-124 rs5315649 had a worse OS than the carriers with Allele G (HR = 1.50, 95% CI: 1.09–2.07, *P* = 0.00). Compared with the carriers with CG + GG genotype of miR-124 rs531564, for the OS, patients with CC showed significant association (HR = 4.61, 95% CI: 1.85–11.49, *P* = 0.00). Patients carrying with Allele C and CC genotype were associated with a poor PFS (HR = 1.67, 95% CI: 1.20–2.33, *P* = 0.00; HR = 2.37, 95% CI: 1.36–4.13, *P* = 0.00) (Table 3).

### Test of Heterogeneity

The results of heterogeneity tests were presented in Table 3. There was no significant heterogeneity between the miR-124 expression (OS, *I*^2^= 21%, *P* = 0.23), methylation (OS, *I*^2^ = 0%, *P* = 0.84; PFS, *I*^2^ = 43%, *P* = 0.17), and polymorphisms (OS, allele, *I*^2^ = 0%, *P* = 0.58; PFS/RFS, allele, *I*^2^ = 0%, *P* = 0.98) and the risk of tumorigenesis, except the expression for PRS/DFS (*I*^2^ = 57%, *P* = 0.02). Therefore, the fixed effects were applied to calculate the pooled HR for miR-124. Meanwhile, meta-regression was applied to investigate sources of heterogeneity for PRS/DFS of expression (Table 4).

**Table 4:** The results of heterogeneity test.

Comparisons	Coef.	SE	*t*	*P*	95% CI
Expression (PRS/disease-free survival)					
Publication year	−0.100	0.707	−0.14	0.900	−3.142 to 2.942
Ethnic*	–	–	–	–	–
Cancer type	0.195	0.353	0.55	0.636	−1.322 to 1.712
Language	−0.161	1.199	−0.13	0.905	−5.318 to 4.996
Assay	−0.351	1.198	−0.29	0.797	−5.508 to 4.808
Sample size	0.844	0.803	1.05	0.404	−2.614 to 4.302
Cut-off	−0.279	1.782	0.19	0.870	−7.334 to 7.996

*Ethnic was dropped because of collinearity.

### Sensitivity Analyses

Sensitivity analyses were carried out to assess the contribution of each study to the pooled estimate. Omitting individual dataset in each comparisons and recalculating did not substantially change the pooled HR, indicating that pooled HRs were quite stable (Figure 3).

### Publication Bias

**Figure 4:**
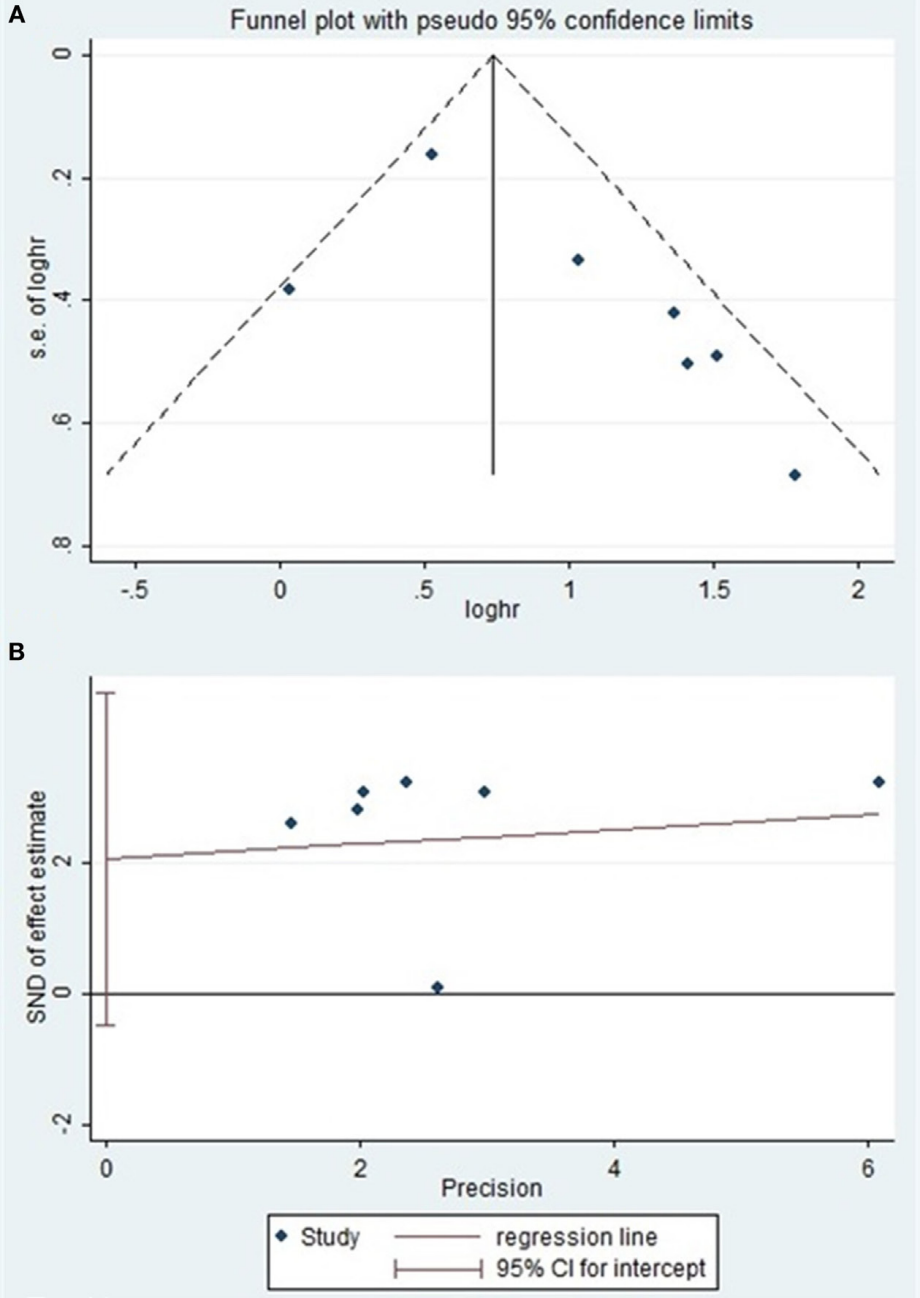
**(A)** Begg’s funnel plot of publication bias on the relationship between miR-124 expression and PRS/disease-free survival (DFS). **(B)** Egger’s funnel plot of publication bias on the relationship between miR-124 expression and PRS/DFS.

Begg’s and Egger’s test were used to evaluate the publication bias. The results suggested no evidence of publication bias (Table 5). Meanwhile, the shape of the funnel plots revealed no visual evidence of the asymmetry (Figures 4A,B).

**Table 5:** Publication bias of miR-17/17-5P for Begg’s test and Egger’s test.

Comparisons	Begg’s test		Egger’s test		
	*z*	*p*	*t*	*p*	95% CI
Expression			1.76	0.107	−0.0599 to 0.5313
Overall survival (OS)-combine	2.14	0.033	5.46	0.000	1.236–2.904
			1.46	0.179	−0.117 to 0.542
Tissue	1.71	0.081	5.09	0.001	1.140–2.964
Serum	−0.34	1.000	0.04	0.975	−11.721 to 11.915
PRS/recurrence-free survival					
Combine	1.57	0.116	1.92	0.097	−0.481 to 4.619
Tissue	1.06	0.288	1.26	0.264	−1.684 to 4.917
Serum	1.04	0.296	1.43	0.389	−77.44 to 97.01
Methylation					
OS	1.02	0.308	1.55	0.261	−1.928 to 4.105
Disease-free survival^a^	0.52	0.602	0.09	0.945	−297.36 to 301.45
Polymorphisms	–	–	–	–	–

*^a^Insufficient observations*.

## Discussion

Emerging studies have indicated that miRNAs could act as oncogenes or tumor suppressors and played key roles in proliferation, differentiation, metastasis, and cell apoptosis of cancer cells (64–66). Therefore, exploring the profiles of miRNAs related to tumorigenesis may promote the understanding of potential mechanisms of cancer development and progression and provide valuable insights for early diagnosis and prognosis of cancer (67, 68).

Several studies have indicated that miR-124 inhibits the epithelial–mesenchymal transition, proliferation, invasion, migration, and angiogenesis of cancer cells (69). However, the association between miR-124 expression, methylation, and genetic variants and cancer survival is still unknown. Therefore, it is very important to address why miR-124 as a prognostic indicator is valuable for judging prognosis and guiding treatment.

In the present study, we revealed that the low expression levels of miR-124 in serum and tissue were significantly associated with poor OS (HR = 2.37, 95% CI: 1.91–2.94, *P* = 0.00 and HR = 3.10, 95% CI: 2.04–4.70, *P* = 0.00) and PFS/RFS (HR = 2.21, 95% CI: 1.50–3.26, *P* = 0.00 and HR = 2.12, 95% CI: 1.20–3.74, *P* = 0.00). We also analyzed the correlation between different methylation levels (OS, HR = 2.09, 95% CI: 1.48–2.95, *P* = 0.00; PFS, HR = 3.70, 95% CI: 1.72–7.97, *P* = 0.00) and SNP (rs5315649) (Allele G: OS, HR = 1.50, 95% CI: 1.09–2.07, *P* = 0.00; Allele G: PFS, HR = 1.67, 95% CI: 1.20–2.33, *P* = 0.00) to evaluate their prognostic significance in cancer patients.

Downregulation of miR-124 has also been observed in various malignancies, including both solid tumors and hematological malignancies (70, 71). It is strictly conservative in both primary sequences and spatial expression patterns, which are limited to the nervous system of different metazoan, including aplysia, nematodes, flies, and all vertebrates studied. This protective effect indicates that miR124 plays an important role in controlling the expression of neural genes (72). Functional studies have linked vertebrate miR-124 to diverse aspects of neural specification or differentiation (73). Dysregulated miRNA expression can be induced by abnormal DNA methylation and contributes to the development and progression of multiple human cancers, including pancreatic cancer (59).

DNA hyper-methylation of miR-124 in pancreatic cancer is mediated by at least part of epigenetic mechanisms (74). Reduced expression of miRNA-124 can be found in pancreatic cancer tissues, and its downregulation was significantly associated with poor OS of PDAC patients. Rac1 as a direct target of miR-124, it has a fundamental role in tumorigenesis and invasion of cancer cells (59).

Epigenetic modifications have been proved to be essential for mammalian development, and epigenetic changes are related to different cancers (75). In cancer cells, some tumor suppressive miRNAs are silenced by the abnormal DNA methylation of CpG islands (76, 77). Therefore, to some extent, aberrant DNA methylation contributes to carcinogenesis and cancer progression.

Polymorphisms of miRNAs can create or destroy miRNA-binding sites and modulate miRNA–mRNA interaction potentially, while those in processing genes can achieve miRNA transcription by altering processing, transcription, or maturation (32). Hsa-mir-124 rs531564 is a relatively consistent predictor of OS, where mutation of each allele can reduce mortality by 30–40% (61). It is a SNP that has been previously found to be associated with the development of cervical cancer, colorectal cancer, and esophageal squamous cell carcinoma (78). Our study bears out this result. In the present study, systematic evaluation was analyzed to precisely quantify the miR-124 expression, methylation levels, and genetic variants. Although our results are robust, following several limitations are worth noting. First, due to not all the included studies reported adjusted HRs and theirs 95% CI, in this case, some data were extracted from survival curves, which could result in several tiny errors. Second, although no evidence of publication bias was found, included studies were mostly in Chinese region, which may generate publication bias. Third, the cut-off values (median, normal mean) were applied to evaluate the different miR-124 expression, methylation levels, and rs531564, which may lead to the deviations of actual values due to different algorithms. Finally, for DFS/PFS, the included studies were not stratified because of the limited availability of eligible studies.

In summary, this is the first study to evaluate the prognostic effects of miR-124 expression, methylation levels, and polymorphisms in different cancer patients. This study showed that low expression and hyper-methylation of miR-124 was strongly associated with poor prognosis, and genetic variations of miR-124 rs531564 affected prognosis in cancer patients. Given its limitations, the results of the study should be interpreted with caution. Future studies are needed to validate these results in prospective studies and evaluate their prognostic role in clinical practice.

## Author Contributions

FD, YS and WL: Conceived and designed the study; YF and JS: Performed the dataset; ZP and LD: Analyzed the data; KW: Contributed analysis and tools material; DF: Wrote the manuscript; WL and YF Reference collection and data management; FD, WL, ZY, and KW: Statistical analyses and paper writing; FD: Study design; YS, revised manuscript.

## Conflict of Interest Statement

The authors declare that the research was conducted in the absence of any commercial or financial relationships that could be construed as a potential conflict of interest. The reviewer HK and handling Editor declared their shared affiliation.

## Appendix

**Table A1:** Quality assessment of included studies based on the Newcastle–Ottawa Scale for assessing the quality of cohort studies.

Study	Selection (score)				Comparability (score)	Exposure (score)			
			
	Representativenes of the exposed cohort	Selection of the non-exposed cohort	Ascertainment of exposure	Outcome of interest was not present at start of study	Based on the design or analysis^a^	Assessment of outcome	Follow-up long enough for outcomes to occur	Adequacy of follow-up of cohorts	Total score^b^
Cong et al. (45)	1	0	1	1	2	1	1	0	**7**
Margolinmiller et al. (46)	1	1	1	1	2	1	1	1	**9**
Liu et al. (47)	1	0	0	1	2	1	1	0	**6**
Sun et al. (48)	1	0	0	1	0	1	1	0	**4**
Ali et al. (49)	1	0	0	1	1	1	1	0	**5**
Dong et al. (50)	1	0	1	1	2	1	1	0	**6**
Li et al. (51)	1	0	1	1	2	1	1	0	**7**
Lv et al. (52)	1	0	1	1	2	1	1	0	**7**
Chen et al. (53)	1	0	1	1	1	1	1	0	**6**
Zhang et al. (54)	1	0	1	1	2	1	1	0	**7**
Jinushi et al. (55)	1	0	0	1	2	1	1	0	**6**
Wang et al. (56)	1	0	1	1	2	1	1	0	**7**
Wang et al. (57)	1	0	1	1	0	1	1	0	**5**
Kim et al. (30)	1	0	1	1	0	1	1	0	**5**
Peters et al. (58)	1	0	1	1	0	1	1	0	**5**
Wang et al. (59)	1	0	1	1	2	1	1	0	**7**
Gebauer et al. (60)	1	0	1	1	1	1	1	0	**6**
Faluyi et al. (61)	1	1	1	1	0	1	1	0	**6**
Shi et al. (62)	1	1	1	1	1	1	1	1	**8**
Ying et al. (63)	1	1	1	1	0	1	0	0	**5**

*^a^When there was no statistical significance in the response rate between case and control groups by using a chi-squared test (*P*˃0.05), one point was awarded*.

*^b^Total score was calculated by adding up the points awarded in each item*.
